# Genome editing and beyond: what does it mean for the future of plant breeding?

**DOI:** 10.1007/s00425-022-03906-2

**Published:** 2022-05-19

**Authors:** Tien Van Vu, Swati Das, Goetz Hensel, Jae-Yean Kim

**Affiliations:** 1 Division of Applied Life Science (BK21 Four Program), Plant Molecular Biology and Biotechnology Research Center, Gyeongsang National University, Jinju, 660-701 Republic of Korea; 2 National Key Laboratory for Plant Cell Biotechnology, Agricultural Genetics Institute, km 02, Pham Van Dong Road, Co Nhue 1, Bac Tu Liem, Hanoi 11917 Vietnam; 3 Centre for Plant Genome Engineering, Institute of Plant Biochemistry, Heinrich-Heine-University, Universitätsstraße 1, 40225 Düsseldorf, Germany; 4 Centre of Region Haná for Biotechnological and Agricultural Research, Czech Advanced Technology and Research Institute, Palacký University Olomouc, 78371 Olomouc, Czech Republic; 5 Division of Life Science, Gyeongsang National University, 501 Jinju-daero, Jinju, 52828 Republic of Korea

**Keywords:** Genome engineering, New plant breeding techniques, CRISPR-Cas, Crop breeding, Biotechnology regulation

## Abstract

**Main conclusion:**

Genome editing offers revolutionized solutions for plant breeding to sustain food production to feed the world by 2050. Therefore, genome-edited products are increasingly recognized via more relaxed legislation and community adoption.

**Abstract:**

The world population and food production are disproportionally growing in a manner that would have never matched each other under the current agricultural practices. The emerging crisis is more evident with the subtle changes in climate and the running-off of natural genetic resources that could be easily used in breeding in conventional ways. Under these circumstances, affordable CRISPR-Cas-based gene-editing technologies have brought hope and charged the old plant breeding machine with the most energetic and powerful fuel to address the challenges involved in feeding the world. What makes CRISPR-Cas the most powerful gene-editing technology? What are the differences between it and the other genetic engineering/breeding techniques? Would its products be labeled as "conventional" or "GMO"? There are so many questions to be answered, or that cannot be answered within the limitations of our current understanding. Therefore, we would like to discuss and answer some of the mentioned questions regarding recent progress in technology development. We hope this review will offer another view on the role of CRISPR-Cas technology in future of plant breeding for food production and beyond.

## Introduction

Crop traits are inherited from parent plants through many generations and are encoded by the genetic information contained within the DNA of cells. However, the genetic contents are continuously modified (Vergauwen and De Smet 2017) as a consequence of spontaneous mutations, transcriptional errors, environmentally or artificially induced mutations, transposon activities, meiotic crossing over, and cross-fertilization (allogamy) processes. Plant pests or symbiotic agents, such as *Agrobacterium* spp. (Kyndt et al. 2015) or *Burkholderia* (Pinto-Carbó et al. 2016), integrate their genetic fragments with the hosts, thereby modulating host cells for replication or feeding. Therefore, genetic modifications continuously occur in any genetic containment of living plant cells.

**Fig. 1:**
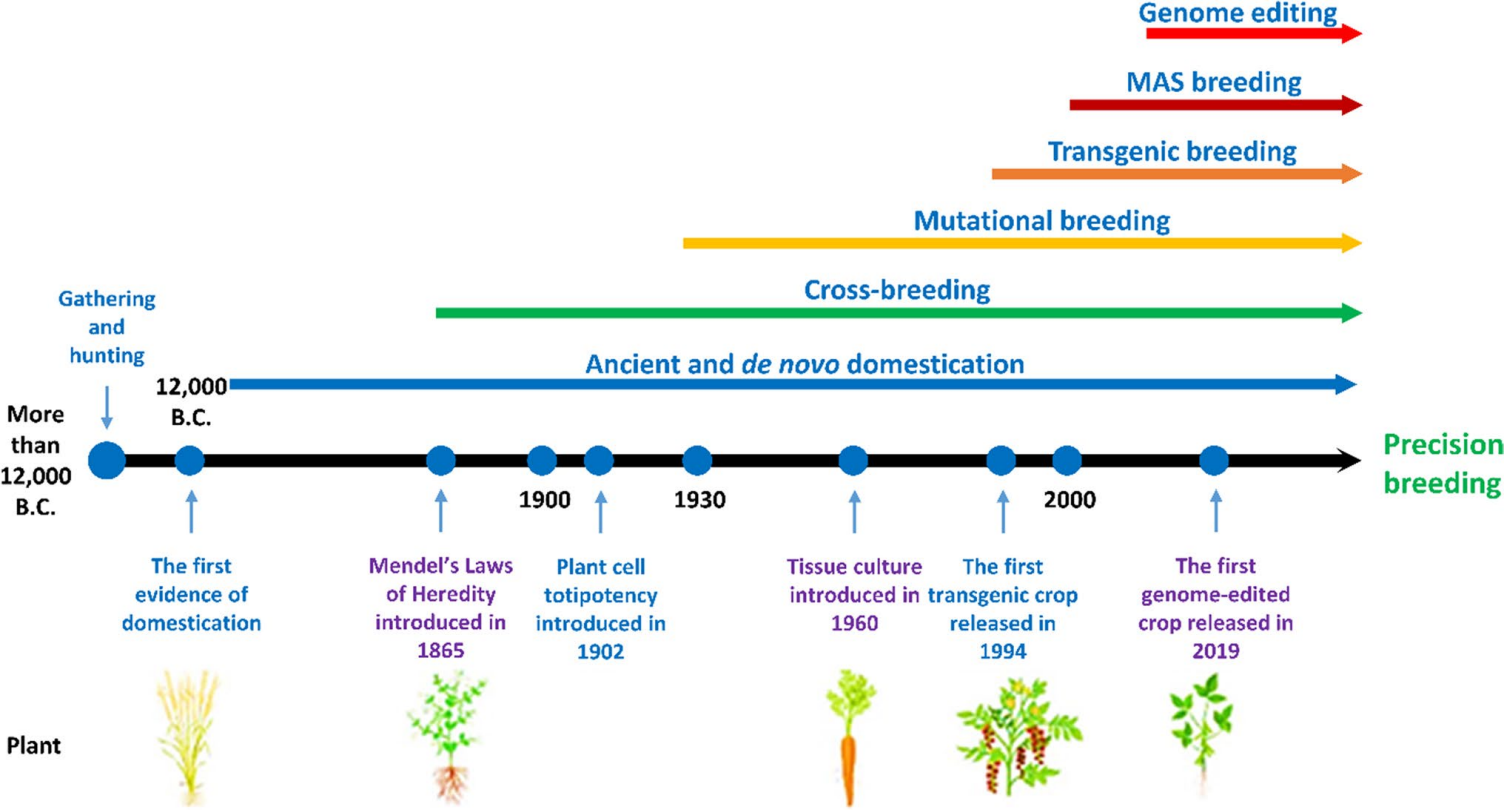
Plant breeding milestones. The start of domestication and initial plant breeding dates back around 12,000 B.C. when the living style of Human-being changed from gathering and hunting to agriculture. The first-ever domesticated plant was emmer wheat. Since then, ancient domestication and selective breeding were dominant until the discovery of Mendel’s laws of genetics. The laws of genetics triggered and enhanced the crossbreeding wave. A milestone in plant breeding that plays an essential role in modern plant breeding was the invention of the totipotency of plant cells in the early 1900s by Gottlieb Haberlandt. As a result, the first in vitro tissue culture was introduced in 1960 with carrot. Plant tissue culture was the critical step for generating the first *Agrobacterium*-mediated transgenic tomato in 1994, known as transgenic breeding. In the meantime, mutational breeding using chemical or physical agents was also introduced in the 1930s and played an important role in generating diverse genetic materials for crop breeding. Biochemical markers further enhanced crossbreeding in marker-assisted selection (MAS) breeding. The recently emerging genome editing approaches have revolutionized plant breeding to precision levels that have never been obtained before. High oleic acid soybean, the first genome-edited crop that was released in 2019, has been opening the wave of genome-edited precision breeding in plants

Plant breeding is the process of generating plants containing genetic entities encoding favorable traits that fit our agricultural production, processing, and subsequent consumption. Thus, it includes selection processes among a population of plants with diverse and undesirable traits. Via archeological evidence, plant breeding has been estimated to have been actively carried out by humans a dozen thousand years ago (Vergauwen and De Smet 2017) when seeds of plants with favorable features were saved for the next plantation, a practice known as domestication. The historical milestones (Fig. 1) of plant breeding techniques were achieved in parallel with a more profound understanding of plants and their genetic makeup. With the increases in quantitative and qualitative food consumption, plant breeding has been revolutionized, with key achievements in crossbreeding (hybrid crops) and transgenesis. Hybrid crop backing by heterosis created the first green revolution, starting with semidwarf wheat varieties carrying *reduced height* (*Rht*) alleles developed by Dr. Norman Borlaug in the 1940s–1950s (Swaminathan 2009; Vergauwen and De Smet 2017). Transgenic crops are now widespread globally and are increasingly accepted as food and feed. In the US, one of the leading countries in adopting transgenic crops, nearly all corn, cotton, and soybean areas are covered by transgenic varieties in 2021 (USDA 2020). Transgenesis changes the genetic information of a plant cell, resulting in a so-called genetically modified organism (GMO) or living modified organism (LMO), by adding "foreign," cross-kingdom DNA fragment(s) to achieve beneficial trait(s) that the conventional breeding techniques cannot obtain. While health and biodiversity concerns regarding transgenic approaches are serious, there is very controversially limited evidence to support them. Nevertheless, a long and expensive procedure is required to release GMO/LMO events for cropping or consuming their products as food or feed, limiting the spreading of crops globally.

Recently, genome editing (GE), especially clustered regularly interspaced short palindromic repeats (CRISPR)-CRISPR-associated protein (Cas), has appeared to offer powerful breeding tools without adding exogenous DNAs producing genetic variations, such as natural, spontaneous mutations. The approach has been shown to create many elite traits that conventional breeding either cannot generate or could only generate with a time-consuming and laborious process (Vu et al. 2020). As a result, GE crops, especially those that carry genome modifications mimicking spontaneous mutations, are widely deregulated worldwide except in European countries where stringent GMO rules are applied (Schmidt et al. 2020b). What makes GE products more acceptable than GMOs? GE is usually triggered using "molecular scissors" to cleave the double-stranded DNA genome. The resulting DNA double-stranded breaks (DSBs) prime cellular responses that lead to the repair of broken DNAs primarily by nonhomologous end-joining (NHEJ) or homology-directed repair (HDR) (Puchta 2005; Scully et al. 2019). DSB repair by NHEJ is dominant in somatic cells and usually results in erroneous products containing short base insertions or deletions, thus inducing mutations to the targeted genes (Puchta 2005). These mutations can be produced naturally as spontaneous mutations or in plants created from chemical/radiation-based random mutagenesis. Notably, the introduced molecular scissors can be eliminated from the edited cells, resulting in transgene-free GE plants, thereby eliminating the concerns associated with the derived foods in some countries (Entine et al. 2021; Menz et al. 2020). Moreover, the edited site is controllable and highly specific due to the characteristics of the customized nucleases (Jinek et al. 2012; Gaj et al. 2013). Therefore, GE products are much "cleaner" than random mutagenesis products.

Under the current conditions of plant breeding and cropping technologies, it is impossible to produce sufficient foods to feed the world by 2050. Additionally, cropping for foods faces many hurdles due to climate change and the shortage of arable lands as a result of urbanization. Therefore, adopting a revolutionized plant breeding technique such as GE is needed to generate new traits/cultivars that can enhance yield or sustain food production under unfavorable conditions. In this review, we discuss the foundation of CRISPR-Cas technology regarding crop genetic improvement and the potential uses of its derived foods for humans compared with other genetic engineering technologies. Future perspectives of GE are also provided to suggest another view of the applied legislation.

In summary, CRISPR-Cas technology enables rapid and precise modification of breeding material, with targets, such as product ingredients, plant architecture, improved nutrient uptake or distribution in the crop, contributing to agricultural sustainability and the UN Sustainable Development Goals (SDGs). Increased yields using less fertilizer, pesticide, or water are further goals of CRISPR-Cas technology.

## Food production in the CRISPR era

According to an analysis performed by van Dijk and coworkers, under the impacts of climate change, global food production needs to be increased from + 30 to + 62% between 2010 and 2050 (van Dijk et al. 2021). If we also consider nutritional needs, then food production may be sufficient in 2050 following radical changes in food types, such as consuming more plant-based alternatives of meat and dairy, and societal adaptation (Berners-Lee et al. 2018), which is likely impossible. Earlier, in the middle of the twentieth century, novel semidwarf wheat and rice varieties played a vital role during the first green revolution (Van Vu et al. 2019), which reversed the catastrophic prediction of hunger due to the food crisis. Food production was dramatically increased in Mexico, India, and other countries following the importation of new wheat and rice varieties (Khush 2001). The breeding technology behind the success of the green revolution was crossbreeding that pyramided elite genes into elite plant varieties, which were selected under various geographical conditions (Swaminathan 2009; Vergauwen and De Smet 2017). Recently, it was predicted that crop-based food production may not be sufficient to feed the world by 2050 (Ray et al. 2013). To reverse the prediction, we must improve crop productivity with high-yield and/or climate-resilient varieties. Hence, novel varieties are expected to play the same role in solving the next food crisis.

GE technology has paved the way for breeding crop plants to enhance growth and production under challenging environmental conditions. It can also help improve crop plants' qualitative traits to sustain human health and other needs. Theoretically, any modification of a known genome site can be made at high efficiency and precision with the various GE tools currently available (Chen et al. 2019; Van Vu et al. 2019; Marzec and Hensel 2019). Early in the development of GE tools, rice varieties that conferred resistance to bacterial blight disease (Li et al. 2012) and fungal blast (Wang et al. 2016) were developed (Table 1). A similar GE tool was also used to create powdery mildew-resistant wheat (Wang et al. 2014). By enhancing yield and improving the starch components in maize, superior corn was introduced in 2020 (Gao et al. 2020). Recently, more attention has been given to the improvement of nutritional and health-functional traits of crop plants, such as oil contents of soybean (Haun et al. 2014), the first commercialized GE product, starch quality in potato (Andersson et al. 2018) and gluten-free wheat (Sánchez-León et al. 2018). Japan was the first country to commercialize a genome-edited tomato product that contains high ɣ-aminobutyric acid (GABA) contents (Nonaka et al. 2017) in September 2021. Increasingly, GE-derived foods and products enhance global food production and security with levels of speed, efficacy, and precision that no other plant breeding technologies can offer. Likewise, with the huge potential of GE technology, global food production can match the projected demands in 2050 in the CRISPR era if the technology is widely accepted in a similar manner to random mutagenesis.

**Table 1:** Several representative CRISPR-Cas-based food crops suggested in this minireview

No.	Crop	Modification	Added trait	Targeted gene(s)	Targeted site	Regulation status	Release status	References
1	Rice	Indel mutation	Bacterial blight resistance	*Os11N3* (*OsSWEET14*)	Cis-element	NA	NA	Li et al. (2012)
2		Indel mutation	Rice blast Resistance	*OsERF922*	Coding sequence	NA	NA	Wang et al. (2016)
3	Wheat	Indel mutation	Powdery mildew resistance	*TaMLO*	Coding sequence	NA	NA	Wang et al. (2014)
4		Indel mutation	Reduced gluten content	*α-gliadin* genes	Coding sequence	NA	NA	Sánchez-León et al. (2018)
5	Maize	Indel mutation	High-yield waxy corn	*ZmWx1*	Coding sequence removal	NA	NA	Gao et al. (2020)
6	Soybean	Indel mutation	High oleic acid oil	*GmFAD2-1A and GmFAD2-1B*	Coding sequence	Passed in the US	February 2019	Haun et al. (2014)
7	Potato	Indel mutation	Altered starch contents	*StGBSS*	Coding sequence	NA	NA	Andersson et al. (2018)
8	Tomato	Indel mutation	Highly accumulated GABA	*SlGAD2* and *SlGAD3*	Coding sequence, autoinhibitory domain removal	Passed in Japan	September 2021	Nonaka et al. (2017)

## What would new plant breeding technology such as CRISPR-Cas-based gene editing offer?

New breeding methods, such as CRISPR-Cas technology, allow rapid and precise manipulation of a target genome for the first time in human history. All previous mutation techniques either involve introducing many unwanted changes (e.g., chemical and radiation mutagenesis) or are purely random and unpredictable (e.g., natural mutations).

In crossbreeding, which is most commonly used for combining positive traits, the genome of the elite background must then be enriched again through several backcrossing steps. Crossbreeding is time-consuming and costly. In addition, this technology has the disadvantage that certain genomic regions cannot be separated from each other due to a lack of recombination. This phenomenon is called linkage drag.

CRISPR-Cas technology can also increase breeding diversity within a species. Here, a reduction in genetic diversity has occurred in the course of domestication due to the processes described above (Voss-Fels et al. 2019). Enhanced genetic diversity can now be achieved through the targeted induction of double-strand breaks between previously inseparable loci (for review, see Rönspies et al. 2021) (Rönspies et al. 2021). Several approaches have described heritable inversions and translocations in this way. Schmidt and colleagues (2020) used the *Staphylococcus aureus* Cas9 nuclease and the ovule-specific promoter in *Arabidopsis* to invert an 18-kb fragment. Subsequently, the same group succeeded in changing the difference between the two *Arabidopsis* ecotypes *Ler*-1 and *Col*-O, known as a heterochromatic knob (*hk4S*) inversion on chromosome 4 (Schmidt et al. 2020a). This inversion made a meiotic crossover in this region on chromosome 4 possible for the first time. Recently, a translocation of 0.5- to 1-Mbp chromosomal segments was reported (Beying et al. 2020). Fragments could be exchanged between *Arabidopsis* chromosomes 1 and 2 and between chromosomes 1 and 5. These changes were also detectable in subsequent generations. Even though similar evidence for crop plants has been lacking, it is now possible to exchange positively correlated alleles between accessions of a species in a targeted manner using the methods described above.

Another advantage of CRISPR-Cas technology is the acceleration of the breeding process. Knowing the molecular basis for domestication genes, such as fruit color, fruit size, or the number of fruits, makes it possible to precisely convert a wild species into a cultivated plant within one generation (for review, see Fernie and Yan 2019) (Fernie and Yan 2019). This process is also referred to in the literature as de novo domestication, and several examples already exist. For example, by altering six loci in the precursor of our present-day tomato *Solanum pimpinellifolium*, plants were generated that had a threefold increase in fruit size, a tenfold increase in the number of fruits, and a 500-fold increase in the accumulation of lycopene (Zsögön et al. 2018). Parallel attempts were made in the same species to alter daylength sensitivity, shoot architecture, flower and fruit production, and nutrient content by simultaneous gene editing (Li et al. 2018). These authors also reported successful domestication in only one step. Similar approaches in *Physalis pruinosa* (Lemmon et al. 2018) and allotetraploid wild rice (Yu et al. 2021) further demonstrated the potential of this technology for the breeding process.

Although many agronomically relevant traits are not based on single modifications, some examples illustrate that this is still possible. The domestication-related barley genes for brittleness and row-type *Btr1*, *Btr2* and *Vrs1* (Komatsuda et al. 2004, 2007) demonstrate that few modifications dramatically change the phenotype. Either the deletion of 1 or 11 nucleotides allows the barley grains to stay in the spike until harvest, which allowed ancient farmers easier harvest (Pourkheirandish et al. 2015). For the switch from two- to six-rowed barley, a mutation in the *Vrs1* gene is necessary. It has been illustrated using RNAi technology that the causal gene has been identified (Sakuma et al. 2013). Using CRISPR/Cas-mediated knockout, full development of the lateral grains was observed (Hensel et al., unpublished). Another example of fungal resistance in wheat further supports the idea that slight modifications have a severe impact. The *Lr34* gene decides whether a wheat accession is susceptible or tolerant to fungal infections. As barley (Chauhan et al. 2015) shows, just one amino acid can alter the resistance of a cultivar. Using CRISPR-Cas technology as prime editing can achieve such allele exchange.

All targeted mutagenesis platforms rely on the knowledge of sequence information. With the tremendous improvements in sequencing technologies, discovering new genomes is possible within hours and for an affordable amount of money. This development allows the direct comparison of multiple accessions of the same species (known as pangenome, (Golicz et al. 2016)) and identifies the beforementioned necessary sequence polymorphisms.

The fundamental prerequisite of the aforementioned accelerated evolution is the simultaneous targeting of multiple target sites in a genome. Various methods have been developed and applied for this purpose. Either several gRNA arrays can be united in one T-DNA using the methods based on type-II restriction enzymes (Hahn et al. 2020), or tRNA-based techniques are used (Xie et al. 2015). Reducing allergens in food is highlighted here from the endless array of applications. First, gluten-free wheat should be mentioned (Sánchez-León et al. 2018). Simultaneously, targeting 35 different *α-gliadin* genes in bread wheat reduced the coeliac disease-triggering gluten content by 85%. This reduction makes the production of gluten-reduced foods possible. Recently, CRISPR-Cas was used to remove the major allergen, 2S albumin class gene *Bra j* I in brown mustard (*Brassica juncea*), and mutant plants with reduced allergenicity were generated (Assou et al. 2021).

The CRISPR-Cas technology application can go far beyond inducing a double-strand break by fusing functional domains to Cas proteins. For example, genes could be activated in rice (Xiong et al. 2021), repressed in *Arabidopsis* (Lowder et al. 2015), their position in the *Nicotiana tabacum* genome visualized by reporter genes (Dreissig et al. 2017), or modified using base editors. Review articles on how these approaches can be used for breeding salt-tolerant rice (Ganie et al. 2021) or tomato (Vu et al. 2020) have recently been published.

A variety of prime editing (PE) and base editing (BE) variants have been developed over the past years and have been successfully tested in plants (for review, see Molla et al., 2021) (Molla et al. 2021). Prime editing (Anzalone et al. 2019) and base editing (Gaudelli et al. 2017; Ren et al. 2018) now allow modification of the target sequence, which does not require induction of a double-strand break. With these, applications, such as regulation of cis-elements, modification of RNA splice sites, integration of synthetic miRNAs, or adaptation of miRNA-binding sites, are possible. This mechanism could also alter the binding sites of effectors released by fungal pathogens to target plant susceptibility genes. In this way, heritable resistance might be conferred.

Using CRISPR-Cas technology, generating resistance to herbicides is also possible. Here, a wealth of herbicide resistance can be produced in agronomically essential plants through the targeted selection of target genes. While most plants for commercial use constitute a GMO through the transfer of bacterial genes, the adaptation of plant targets, such as *EPSPS*, *HPPD*, and *ALS* genes from *Zea mays*, *Avena sativa*, and *N. tabacum*, can be made using CRISPR-Cas technology. This review provides a detailed overview (Han and Kim 2019).

## What makes CRISPR-Cas the most powerful gene-editing technology?

CRISPR-Cas technology, unlike zinc finger nucleases (ZFNs) and transcription activator-like effector nucleases (TALENs), is an RNA-based gene modification system (Jinek et al. 2012). ZFNs and TALENs, in contrast, are based on the attachment of polypeptides to DNA. In ZFN, a finger-like peptide binds a DNA triplet (Urnov et al. 2010), whereas, in TALEN, approximately 33 amino acid-length peptides bind specifically to a DNA nucleotide (Boch et al. 2009). In ZFN and TALEN, these DNA-binding domains are coupled to the nonspecific FokI endonuclease. However, this is only active as a dimer (Vanamee et al. 2001), which means that ZFN and TALEN approaches require two target molecules on the respective complementary strand. This mode of action makes cloning more difficult because larger and repetitive DNA fragments have to be assembled. The target sequence area is thus larger, which increases the specificity and reduces the probability of an off-target mutation. The basis for this is that a DNA region of approximately 40 nucleotides per target genome is more likely to be unique than a region only 20 nucleotides long, as with CRISPR-Cas. However, this potential negative feature of the CRISPR system can be reduced by carefully selecting the target sequence. Various online tools, such as CRISPOR (Concordet and Haeussler 2018) and CRISPR-Plant (Xie et al. 2014), will only be mentioned here as examples. A review on this has been published previously (Cui et al. 2018; Naim et al. 2020).

Proof of the development of revolutionary technology is its broad application. Apart from the introduction of next-generation sequencing technology, there has been no other technology in the life sciences in recent years that has been used so widely and successfully in such a short time. The reason for this is its universal use in every organism tested to date, but above all, its simplicity in creating the necessary reaction reagents. For the most common usage, the transformation of a simple vector carrying expression cassettes encoding a CRISPR-Cas protein and a guide RNA (gRNA) is sufficient in many cases. Additionally, the creation of complex constructs is straightforward through the implementation of methods based on type-II restriction enzymes (Engler et al. 2008). Furthermore, in recent years, commercial suppliers have CRISPR products in their portfolios at affordable prices, further fueling the use of CRISPR-Cas technology.

As CRISPR-Cas technology uses a relatively short sequence (on average, 20 nucleotides) to navigate the double-strand inducing Cas protein, special attention must be paid to gRNA selection. Multiple online platforms allow the identification of target genome-specific sequences (Liu et al. 2020). It has to be mentioned that not just sequence similarities of the gRNA are essential. The necessity of being adjacent to a PAM sequence further reduce putative off-targets (Jinek et al. 2012). Several studies have shown that mismatches in the twelve nucleotides in front of the PAM abolish the functionality of the RNP complex (Zheng et al. 2017). Another possibility to reduce potential off-targets is using preassembled RNP complexes (Park and Choe 2019). As these complexes do not contain DNA that becomes integrated into the host genome and is transmitted during cell divisions, inducing off-targets is reduced.

The biotechnological use of the CRISPR-Cas system involves two components. These are target sequence-specific gRNAs and double-strand break-inducing Cas enzymes (Jinek et al. 2012). The Cas enzyme to bind to the target DNA requires a so-called protospacer adjacent motif (PAM) sequence. This PAM sequence is specific for each Cas protein. For the most commonly used SpCas9, NGG somewhat limits the choice of a target sequence. Although there are sufficient NGG positions in each genome, if one needs to cut at a specific place due to a functional amino acid or protein domain being present, this can lead to limitations. This disadvantage has been overcome by identifying Cas proteins from other organisms or the synthetic evolution of existing Cas proteins (for review, see Liu et al. 2021) (Liu et al. 2021).

Another advantage of the CRISPR-Cas system is its DNA-binding properties. By inactivating the catalytic domains, activity-killed Cas protein can target specific areas of a target genome. This approach can be used for gene activation (Xiong et al. 2021), repression of gene function (Lowder et al. 2015), visualization of specific genomic regions (Dreissig et al. 2017), or setting epigenetic marks (Nakamura et al. 2021). This diversity of applications demonstrates the broad and universal use of a modified bacterial immune system (Huang and Puchta 2021).

In summary, CRISPR-Cas technology has revolutionized the life sciences as a whole, not only basic biology or agriculture. Many applications use the technology in medicine as exemplified by diagnostics (Hajian et al. 2019) or altering cancer treatments (Stadtmauer et al. 2020). It is one of the most powerful technologies discovered recently.

## What are the differences between CRISPR-Cas and other genetic engineering techniques?

### The era of conventional plant breeding methods

Since the Neolithic age, plant breeding has been a paramount part of an evolving human civilization. It is based on the introduction of genetic variability, yield increment, and improved nutritional value in crops by crossing wild species and landraces (Fig. 1) (Podevin et al. 2013). However, in this contemporary age of agriculture, conventional breeding techniques, such as crossbreeding and mutagenesis breeding, using irradiation or chemical mutagenesis are unlikely to be adapted to mass production to feed approximately 10 billion people by 2050 (FAO 2017; Gao 2021). In the late 1950s, the first Green Revolution, introducing “dwarfing” gene mutations, was bred into major staple crops, such as wheat (*Triticum aestivum*) and rice (*Oryza sativa*), to obtain high-yield crops (Khush 2001). In chemical and radiation-induced mutation breeding, genome-wide random mutagenesis was observed, which led to genetic variation (Holme et al. 2019). However, both the crossbreeding and mutation breeding techniques encountered limitations, such as introducing undesirable traits, high labor requirements, and the amount of time needed to choose a rare variety that harbored the desired feature (Gao 2021).

The long history of plant breeding pivoted its focus from crossing, random mutagenesis, and transgenic breeding to genome editing (Hickey et al. 2019; Chen et al. 2019). Modern plant breeding techniques can be one of the critical solutions for securing food production to feed the world under emerging biotic and abiotic risks, such as environmental and climatic changes and disease and pest management (Podevin et al. 2013).

### Transgenic breeding and new plant breeding techniques

Transgenic breeding opened a new era, but its products contain foreign DNA fragments with random insertions in the genome that may cause potential unintended effects. New plant breeding techniques were introduced to overcome some of these transgenic products. These technologies include cisgenesis and intragenesis, grafting, agroinfiltration, reverse breeding, RNA-directed DNA methylation, genome elimination, oligonucleotide-directed mutagenesis (ODM), and site-directed nucleases (SDNs) (Enfissi et al. 2021). However, most of them could not overcome the regulatory limitations in many countries (Podevin et al. 2013). Cisgenesis and intragenesis can be categorized under a traditional genetic transformation technology of plant breeding, as they involve T-DNA integration into a plant genome. Intragenesis creates new genes with desired traits by isolating functional genetic elements, such as promoters, coding regions, or terminators of existing genes, rearranging them in vitro, and inserting the ‘intragenic’ DNA combination back into the plant (Rommens et al. 2007). In contrast, a ‘cisgenic plant’ is a crop plant that has been genetically modified with one or more genes (containing conserved introns and flanking regions, such as native promoter and terminator regions in a sense orientation) isolated from a crossable donor plant (Schouten et al. 2006). Cis/intragenic lines should be free from foreign sequences (i.e., selectable markers and reporter genes) (Liu et al. 2013).

The use of *Agrobacterium tumefaciens*-derived T-DNA borders is a particular concern for public acceptance in GMO regulation; thus, it can be preferentially replaced by plant-derived P-DNA borders (Schouten and Jacobsen 2008). The debate for using P-DNA borders for *Agrobacterium*-mediated transformation over T-DNA borders is that DNA sequences integrated into the recipient plant should be derived from the sexually compatible DNA pool (Rommens et al. 2004). However, in the case of T-DNA sequences, it can be identified within different plant species, as sometimes it integrates into the genome without integration of T- DNA border sequence, thus making an alternative way to identify and select transformants (Zhu et al. 2013).

Nevertheless, this technique randomly integrated foreign DNA into plant genomes and was strictly subjected to government regulations for introducing foreign genes; the products were called GMOs (genetically modified organisms). To date, public and government opinions remain largely undecided regarding the safe usage of the final products created by cisgenesis and intragenesis approaches (Raman 2017; Gao 2021). Apart from transgenic breeding that maintains inserted genes, another class of NPBTs temporally introduces recombinant genes that change the expression of one or more endogenous genes, resulting in reverse breeding or early flowering. Another extended method is RNA-directed DNA methylation for gene silencing induced by transient expression via agroinfiltration or grafting to a GM stock (Bally et al. 2018; Enfissi et al. 2021). In the absence of an introduced recombinant gene or removal by segregation, these techniques result in no change in their native genome in the final products (Schaart et al. 2016).

### Plant genome editing

The era of gene editing in NPBT makes precise manipulation of the genome possible with SDNs (Table 2), which are primarily executed as molecular scissors of the genome by programmable DNA-binding proteins that recognize a specific DNA sequence of a DNA target and induce DSBs (Gaj et al. 2013; Shrivastav et al. 2008; Enfissi et al. 2021). Although uncertainties remain over the future applications of plant genome editing, this technique has the potential to surpass the time-consumption and regulatory limitations associated with conventional (crossing and random mutagenesis) and transgenic breeding techniques, respectively (Lusser and Rodríguez-Cerezo 2012). The advantage of gene editing over genetic engineering is that the end product acquires no foreign genes (Schaart et al. 2016). The associated plants produced via the SDN1 method are designed to introduce random mutations (substitutions, insertions, and deletions) using double DSBs to a specific gene, followed by a NHEJ repair pathway (Roth et al. 2012; Waterworth et al. 2011). Deletions of regulatory regions, exons, introns, or large chromosomal fragments by introducing one or two DSBs at different sites using either one or two SDNs lead to frameshifts, duplication, inversion, and translocation events (Petolino et al. 2010; Şöllü et al. 2010; Lee et al. 2012; Fauser et al. 2012). SDN2-edited products are developed in the presence of the donor DNA repair template containing targeted mutations by single-base substitution or short indels and have high sequence identity to the endogenous gene by exploiting HDR pathway (Podevin et al. 2013; Schaart et al. 2016). The final edited products from SDN3 can be maneuvered for targeted gene correction or to introduce gene/allele replacement for creating new phenotypes. SDN3 defines an insertion of new DNA fragments at a predefined locus using sequence-specific donor DNA templates with flanking DNAs showing homology to the target locus by both NHEJ and HDR, which would otherwise integrate randomly in the genome at naturally occurring DSBs (Naegeli et al. 2020; Podevin et al. 2013). The ODM approach does not rely on exogenous nucleases but uses oligonucleotides to introduce targeted mutations in the genome, usually of one or a few adjacent nucleotides, an approach that is distinct from SDN-based techniques (Zhu et al. 2000).

**Table 2:** Examples of SDN-1, SDN-2, and SDN3 in plant genome editing

Product type	SDN tool	Application	Plant	Target gene	References
SDN-1	TALEN	Bacterial leaf blight resistance caused by *Xanthomonas oryzae* pathovar *oryzae* in Rice	*Oryza sativa*	Partial deletion (5 to 10 bp) of a specific region in the *Os11N3* gene	Li et al. (2012)
	TALEN	Resistance against *Blumeria graminis* f.s.p. *tritici* (*Bgt*) in hexaploid Bread wheat	*Triticum aestivum*	S-gene disruption of *Mildew resistance locus o* (*Mlo*) gene; six *Mlo* alleles	Wang et al. (2014)
	TALEN	Improving food quality by converting oleic acid to linoleic acid in Soybean (*Glycine max*)	*Glycine max*	*FAD2-1A* and *FAD2-1B*	Haun et al. (2014)
	TALEN	Accumulation of reducing sugars during cold storage in tetraploid commercial potato variety	*Solanum tuberosum*	Four alleles of *Vacuolar invertase*﻿ (*Vinv*)	Clasen et al. (2016)
	CRISPR-Cas	Increased Brassinosteroid signaling by targeting a negative regulator in BR signaling pathway of Lettuce (*Lactuca sativa*)	*Lactuca sativa*	*Brassinosteroid insensitive 2* (*BIN2*)	Woo et al. (2015)
	ZFN	*Arabidopsis* stress-response regulator *ABA-INSENSITIVE *(*ABI4*) gene, by customed designed ZFN	*Arabidopsis thaliana*	*ABA-INSENSITIVE* (*ABI4*)	Osakabe et al. (2010)
	ZFN	High frequency targeted mutagenesis in *Arabidopsis* *thaliana* using zinc finger nucleases	*Arabidopsis thaliana*	indel mutation in *ADH1* and *TT4*	Zhang et al. (2010)
	TALEN	Custom TALEN design for *Arabidopsis* ADH1 target gene using functional analog of *AvrHah1*	*Arabidopsis thaliana*	indel mutation in *ADH1*	Cermak et al. (2011)
	CRISPR-Cas	Bacterial blight susceptible genes in Rice	*Oryza sativa*	*OsSWEET14* and *OsSWEET 11*	Jiang et al. (2013)
	CRISPR-Cas	*AtPDS3* and *AtFLS2* targeted for indel mutation	*Arabidopsis thaliana*	*AtPDS3* and *AtFLS2*	Li et al. (2013)
	CRISPR-Cas	*iInositol oxygenase* (*inox*) and *phytoene desaturase* (*PDS*) gene using cell suspension culture of wheat	*Triticum aestivum*	*inox* and *pds* indel mutation	Upadhyay et al. (2013)
	TALEN and CRISPR-Cas	ZmPDS, ZmIPK1A, ZmIPK, ZmMRP4 using TALEN and *ZmIPK* gene using CRISPR-Cas in maize	*Zea mays*	*ZmPDS, ZmIPK1A, ZmIPK, ZmMRP4*	Liang et al. (2014)
	TALEN	*Vacuolar Invertase* (*VInv*) breaks down sucrose to glucose and fructose in Potato	*Solanum tuberosum*	*VInv*	Clasen et al. (2016)
SDN-2	ODM	Tolerance to imidazolinone herbicides in Maize	*Zea mays*	Acetohydroxyacid synthase gene (*AHAS*)	Zhu et al. (2000)
	ZFN	The *gfp* coding region was replaced by the *hpt* coding region and the *ppt* coding region, in *Arabidopsis*	*Arabidopsis thaliana*	*gfp*- coding region	de Pater et al. (2009)
SDN-3	ZFN	Maize with reduced phytate content and herbicide tolerance	*Zea mays*	*Inositol-1,3,4,5,6-pentakisphosphate 2-kinase* (*IPK*)	Shukla et al. (2009)
	TALEN	Targeted replacement of 322-bp donor molecule differing by 6 bp from *ALS* coding region in *N. benthamiana*	*Nicotiana benthamiana*	gene target replacement in *ALS*	Zhang et al. (2013)
	CRISPR-Cas	*NbPDS* locus designed with donor template containing AvrII site flanked by a 533-bp left homology arm and 114-bp right homology arm	*Nicotiana benthamiana*	HDR in *NbPDS*	Li et al. (2013)

The products of NPBT developed as SDN-1, SDN-2, and SDN-3 (Table 2) are implemented using molecular scissors, such as protein-guided zinc finger nucleases (ZFNs, the first generation of editing tools) (Bitinaite et al. 1998; Laity et al. 2001; Urnov et al. 2005; Kim et al. 1996) and transcription activator-like effector nucleases (TALENs, the second generation) (Miller et al. 2011; Li et al. 2011). The third generation of molecular scissors is an RNA-guided protein complex, CRISPR-Cas systems. The most widely used CRISPR-Cas systems are Cas9 and Cas12a (Cpf1) (Jinek et al. 2012; Zetsche et al. 2015; Kim et al. 2016).

The GE tools ZFN, TALENs, and the CRISPR-Cas system can be used as SDN-1 (for random mutagenesis), SDN-2 (predicted mutagenesis of a targeted genomic locus), and SDN-3 (precision insertion of a DNA sequence). However, the ODM technique resembles the SDN-2 type. Recent GE findings of base editing and prime editing (Komor et al. 2016; Anzalone et al. 2019; Lin et al. 2020) generate specific base changes in the target sequence without inducing DSBs or positioning any template DNA in the targeted locus. SDN tool delivery can be executed in plant genome editing by stable DNA integration, transient expression, or ‘DNA-free’ methods. The SDN modules integrated into the genome can be removed by segregation in edited progeny events in sexually propagated crops. In asexually (vegetatively) propagated crops, transient expression or DNA-free delivery of the SDN tool is required (Ma et al. 2017). For both ODM chemically synthesized oligonucleotide and ‘DNA-free’ delivery, RNA expressing the SDN module or the protein (TALEN, ZFN) or ribonucleoprotein complex (in case of CRISPR-Cas) itself can be delivered directly into plant cells (Metje-Sprink et al. 2018; Okuzaki and Toriyama 2004). In summary, CRISPR-Cas technology provides the most efficient tools to modify endogenous gene sequences precisely, resulting in foreign gene-free GE products.

## Would its products be labeled as "conventional" or "GMO" or something else?

There is a continuous debate regarding the acceptance of GMO/LMO products as foods or feeds (Teferra 2021). The tendency is to assure no potential harm can be caused to consumers' health or no environmental threat. One of the primary reasons for the regulation of GMOs is that cross-kingdom genetic materials are integrated into plant genomes and overexpressed during plant growth and development (Teferra 2021). However, it is different in the case of GE plants, when in some cases, indel mutations are indistinguishable from wild plants since there are no foreign genetic materials integrated into edited plant genomes (Wolt et al. 2016). Therefore, GE plants and derived products are as readily accepted as foods and feed as those obtained from conventional breeding if they do not contain exogenous genetic materials (Entine et al. 2021; Menz et al. 2020). Rapid applications and relaxed regulations have accelerated the release of the first (high oleic soybean) and second (GABA tomato) GE products in less than eight years from the first publication of TALEN and CRISPR-Cas9 techniques, respectively. Significantly, the commercialization of the GE products is much less expensive than that of GMO products thanks to the omission of the costly regulatory process that applied to GMO products. Thus, for the first time, small enterprises, such as Calyxt or Sanatech Seed Co., Ltd, have released new biotech products. Many requests for the deregulation of GE events were from small or medium enterprises (https://www.aphis.usda.gov/aphis/ourfocus/biotechnology/am-i-regulated/regulated_article_letters_of_inquiry/regulated_article_letters_of_inquiry, accessed on 30.11.2021). These facts encourage other small-scale labs and companies to develop new GE crops, thereby bringing hope to the idea that global food production can feed the world by 2050. This section analyzes the two GE cases to understand how they were deregulated by the US and Japan.

### Case #1: GABA tomato

Gamma-aminobutyric acid (GABA) is an amino acid that is not used to make proteins. Consumption of GABA may help to reduce blood pressure and may have several other sound health effects. GABA is an intermediate product of a biochemical pathway called the GABA shunt that converts glutamate to succinate (Bown and Shelp 1997). In plants, GABA accumulation was enhanced under stresses that stimulate the Ca^2+^/calmodulin-dependent activities of glutamate decarboxylase (GAD, EC 4.1.1.15). Suppressing *GAD* genes by RNAi reduced GABA accumulation in plants (Takayama et al. 2015). GAD's C-terminal calmodulin-binding domain (CaMBD) plays an autoinhibitory function in switching GAD activities between normal and stress conditions. Thus, removing it enhanced the GABA contents in tomatoes (Takayama et al. 2017). CRISPR-Cas-based targeted mutagenesis of *GAD2*, *GAD3*, and *GABA transaminase*-encoding genes (*GABA-T*) resulted in hyperaccumulation of GABA in tomato (Sicilian Rouge CF cultivar) (Nonaka et al. 2017). The line containing a mutation that eliminated the CaMBD of GAD2 was the first commercial GE tomato globally.

Sanatech Seed Co., Ltd., the owner of the high GABA line, sent a letter to the Animal and Plant Health Inspection Service (APHIS, USA) to request the exclusion of the high GABA tomato from the APHIS oversight under 7 C.F.R. Part 340 article. It is a T2 segregated line validated as T-DNA-free by conventional PCR covering the entire binary plasmids used for* Agrobacterium*-mediated transformation. The edited allele contains a single-base insertion resulting in a premature stop codon just before the CaMBD domain, eliminating it from the GAD2 protein. Therefore, the line accumulated GABA at levels 4- to fivefold higher than in its parents, and the traits were stable for at least three generations (T0, T1, and T2) (https://www.aphis.usda.gov/biotechnology/downloads/reg_loi/20-140-01_air_CBIdel_a2.pdf, accessed on 29.11.2021). More importantly, the high GABA content does not affect the growth and development of the line. A steroidal glycoalkaloid, tomatine, and its contents in ripened fruits were not altered, indicating that toxin or allergen production does not increase. Ultimately, the high GABA tomato line was confirmed to be deregulated from APHIS oversight under 7 C.F.R. Part 340 in a process called "Am I Regulated (AIR)" (https://www.aphis.usda.gov/biotechnology/downloads/reg_loi/20-140-01_air_response_signed.pdf, accessed on 29.11.2021).

### Case #2: high oleic acid soybean

The GE event of soybean that increased oleic acid content was the first GE soybean to be released to the US market by Calyxt (previously known as Cellectis Plant Sciences). On May 5, 2015, APHIS issued a letter to Cellectis Plant Sciences for deregulating a TALEN-based FAD2KO soybean that accumulated high levels of oleic acid from the 7 C.F.R. Part 340 (https://www.aphis.usda.gov/biotechnology/downloads/reg_loi/brs_response_cellectis_air_fad2k0_soy_cbidel.pdf, accessed on 30.11.2021). The FAD2KO event contains knockout alleles of the *Fatty acid desaturase 2-1a* (*FAD2-1a*, a 63-bp KO allele) and *FAD2-1b* (a 23-bp KO allele) genes, which play roles in converting oleic acid to linoleic acid (Haun et al. 2014). Seeds collected from plants carrying homozygous knockout alleles of the two genes increased the oleic acid content up to 80% compared to 20% in its parental lines. They reduced the linoleic acid content to under 4% (Haun et al. 2014). In the US, the cropping area of FAD2KO has dramatically increased since 2018 (Menz et al. 2020). The event was confirmed not to contain T-DNA by PCRs that amplified three distinct regions of the T-DNA, including the TALEN gene, the selection marker, and the right border (Haun et al. 2014).

## Would production be sufficient with gene-edited varieties?

It is difficult to answer this question since better future technologies can replace the present. Moreover, if the world population continues to increase at the current rate, we will need more improved crop cultivars created by diverse technologies than just GE plants. However, at present, GE varieties are bringing hope to sustain and secure global food production to meet the demand by 2050 since the technology can quickly generate or domesticate desirable alleles into elite cultivars at low costs and with no linkage drag. Nevertheless, GMO/LMO varieties still play essential roles, featuring crucial traits that GE has not been able to successfully generate, such as those that require a strong expression of genes, such as herbicide- or insect-resistant alleles. Usually, genome editing is sufficient to introduce herbicide-resistant alleles into a plant. However, the expression levels, driven by endogenous promoters, might not be adequate to support durable herbicide resistance at high doses while maintaining enzymatic activities (Li et al. 2016; Jin et al. 2021; Hummel et al. 2018). Alternatively, the endogenous promoter can also be engineered by the CRISPR-Cas system to increase its strength (Rodríguez-Leal et al. 2017), which may further advance the technology for those traits. For insect resistance, it seems that only cross-kingdom genes have been successful at this point; hence, insect-resistant GMO/LMO varieties are still needed.

## Future perspectives

Several significant threats endanger global food security, especially in developing countries (Khush 2001; Ray et al. 2013). The first is the decrease in arable lands due to urbanization, which is greatly extended in developing countries. Second is the shifting of environmental conditions caused by climate changes that can affect the sustainability of plant-based food production because the growth and development of plants are sensitive to subtle changes in environmental conditions. The third is the yield barrier limiting conventional breeding techniques from achieving higher productivity. More importantly, the three significant threats become more serious when combined. In the meantime, the global population in developing countries is increasing at speeds much higher than that of food production, threatening developing countries with hunger and undernourishment. Therefore, the UN's Sustainable Development Goals to "end hunger by 2030" may not be reached in time.

All plant breeding techniques have significantly contributed to global food production regardless of the legislation applied to them or the products derived from performing these techniques. There is a clear trend of technological advances in plant breeding and food productivity, indicating their importance. However, when technologies are advanced, new components added to the traditional system cause concerns about potential risks to consumers' health. Therefore, even when proven to be safe for humans, GMO/LMO products are still subjected to costly and lengthy regulation procedures (Teferra 2021). However, transgene-free GE products cannot be more "traditional" and much "cleaner" than products obtained by random mutagenesis. There are even difficulties distinguishing a GE plant from its parents because legislators cannot find any convenient detection tools (Grohmann et al. 2019). Then, the question is, do we have to consider GE plants as riskier than their parents? The answer is no from the leading countries in biotechnological innovation, such as the US and Japan, where the products of the first GE crops were commercialized like traditional ones. Many countries have now deregulated the GE crops listed in the SDN-1 category (Table 2) (Van Vu et al. 2019; Menz et al. 2020). Surprisingly, GE plants have been listed along with GMOs/LMOs in Europe, where cropping of GMOs/LMOs is not allowed, even though these regions are major importers of GMO/LMO products. Fortunately, scientists are raising voices to defend GE plants and their products; for example, the EU-based petition called "give CRISPR a chance" (Vangheluwe et al. 2020) has successfully changed the minds of legislators to reconsider regulatory bills for GE plants (https://www.reuters.com/world/europe/eu-calls-rethink-gmo-rules-gene-edited-crops-2021-04-29/, accessed on 03.12.2021). Moreover, after Brexit, the UK considered genome editing as a science innovation and opened the door for its use (https://www.nature.com/articles/d41586-021-01572-0. Accessed on 03.12.2021).

Although the first concern about a new biotech product is always consumer safety, overestimating their risks in a nonscientific manner has been preventing innovations in plant breeding and food production (Jorasch 2020; Whelan et al. 2020). It may not be a significant issue to wealthy countries like those in the European zone but may affect food security in developing countries. Therefore, giving CRISPR a chance allows developing countries to ensure adequate food production, which will help reach the UN's Sustainable Development Goals to "end hunger by 2030" and further overcome the tragic prediction of food crises in 2050.

## Concluding remarks

Plant breeding has played a critical role in securing food production to feed the world. Regardless of the breeding technique applied to generate a new variety, safety assessments based on the product and not the technology used should be used to determine whether the plant should be released into the environment. Many crop cultivars obtained by random mutagenesis were released into the environment without any restriction simply because they have an extended historical profile of safe use as foods and feeds (Holme et al. 2019). If everything is decided in a science-based manner, then most GE plants should also be considered at least as safe as those obtained from random mutagenesis (Entine et al. 2021; Jorasch 2020; Whelan et al. 2020). In other aspects, compared with GMO/LMO plants, many GE traits can be generated without the incorporation of transgenes. GE plants are likewise very similar to those that evolved in nature but require only months to obtain compared to multiyear selection on multiple generations of ancient domesticated crops. Therefore, it is natural not to regulate GE plants, as shown by Sanatech's high GABA content and Calyxt's FAD2KO events. GE technology is continuously improving, and we are expecting more exciting applications in crop breeding and the global acceptance of GE plants and foods for ending hunger and poverty in every country in the world.

### *Author contribution statement*

TVV, GH, and JYK: Conceptualization; TVV, SD, GH, and JYK: Writing – Original Draft; TVV, GH, and JYK: Writing – Review & Editing; TVV, GH, and JYK: Funding Acquisition; and TVV and JYK: Supervision.

## Abbreviations

CRISPR: Clustered regularly interspaced short palindromic repeat
Cas: CRISPR-associated protein
GE: Genome editing
GMO: Genetically modified organism
LMO: Living modified organism
ODM: Oligonucleotide-directed mutagenesis
SDN: Site-directed nuclease
DSB: Double-stranded DNA break
NHEJ: Nonhomologous end-joining
HDR: Homology-directed repair
